# Genetic characterization of HIV-1 subtype G envelope sequences by single genome analysis

**DOI:** 10.1186/1742-4690-9-S2-P152

**Published:** 2012-09-13

**Authors:** E Rene Ghislain, M Tongo, E Ngolle, W Burgers, J Dorfman

**Affiliations:** 1 International Centre for Genetic Engineering and Biotechnology, Cape Town, South Africa; 2 Division of Medical Virology, University of Cape Town, Cape Town, South Africa; 3 Centre de Recherche en Maladies Emergentes et Reemergente (CREMER), Yaounde, Cameroon

## Background

Subtype G is the sixth most prevalent subtype of HIV-1 and is responsible for an estimated 1,500,000 infections worldwide. Although systematic analyses of a wide range of HIV-1 envelope sequences and neutralization have been performed, subtype G viruses are severely underrepresented in these studies. There is thus an important need to study subtype G envelope sequences and their neutralization capacities.

## Methods

64 Plasma samples from Cameroon were used and 6 were found to be Subtype G by sequencing of gag and nef including one typed only for gag. Single genome analysis (SGA)-PCR was then performed and full length envelope genes were generated, which were then sequenced in the V1-V5 region.

## Results

Phylogenetic analysis of V1-V5 envelope sequences confirmed that 5 samples grouped within the expected subtype G and 1 with subtype A which we had been unable to type for nef. Sequences from within one sample were genetically related to each other, while samples were genetically distinct from each other. This suggests that the sequenced HIV-1 from any donor were generally from a single infection. Sequences from 4 of 5 samples grouped most closely to other West African subtype G sequences, while the fifth grouped in a cluster populated by sequences from Spain and few other African sequences. The V1-V5 region of the sixth sample clustered with subtype A and is thus presumed to be a recombinant.

## Conclusion

These results suggests that our samples capture a substantial amount of the diversity within the subtype G envelope sequences and are largely different from each other. Therefore, forthcoming data concerning the neutralization patterns of these viruses will be able to give some of the sense of the diversity of neutralization pattern of subtype G viruses that will be needed to design a truly global vaccine.

